# Status report on the atopic dermatitis registry TREATgermany 

**DOI:** 10.5414/ALX02262E

**Published:** 2021-08-27

**Authors:** Doreen Siegels, Eva Haufe, Luise Heinrich, Thomas Werfel, Stephan Weidinger, Jochen Schmitt, the TREATgermany Study Group

**Affiliations:** 1 Center for Evidence-Based Healthcare, University Hospital Carl Gustav Carus and Carl Gustav Carus Faculty of Medicine, Technische Universität Dresden,; 2 Department of Immunodermatology and Experimental Allergology, Department of Dermatology, Allergology and Venereology, Hannover Medical School, Hannover, and; 3 Department of Dermatology, Venereology and Allergology, Schleswig-Holstein University Hospital, Kiel Campus, Kiel, Germany; a ORCID ID: 0000-0002-4049-9120,; b ORCID ID: 0000-0001-6830-7672,; c ORCID ID: 0000-0003-3944-252X,; d ORCID ID: 0000-0003-0264-0960

**Keywords:** atopic dermatitis, atopic eczema, clinical symptom severity, clinical registry, systemic treatment

## Abstract

Background: The TREATgermany registry collects data from children, adolescents, and adults with moderate-to-severe atopic dermatitis (AD) in Germany. For this purpose, clinical and patient-reported outcomes, the course of the disease, and applied therapies are observed. Methods: TREATgermany recruits patients with moderate-to-severe AD according to the diagnostic criteria of the UK Working Party, an “Objective Scoring for Atopic Dermatitis” (oSCORAD) > 20 and/or currently antiinflammatory systemic treatment for AD or previous anti-inflammatory systemic treatment for AD within past 24 months before inclusion. No study related interventions will be performed. Currently, 59 dermatological practices, clinics, and university hospitals are participating in TREATgermany (as of May 2021). Based on the interim analysis of October 13, 2020, patient characteristics were described from 4,373 documented visits of adult participants (n = 1,025). Results: The mean age at inclusion in TREATgermany was 42 years, 57.7% of patients were men (n = 591) and 42.3% were women (n = 434). According to oSCORAD, 85.8% of those included suffered from moderate-to-severe AD. At baseline visit, 744 patients had already received one or more systemic treatments for AD (glucocorticosteroids n = 600, ciclosporin A (CSA) n = 307, dupilumab n = 98). 597 patients received dupilumab during their participation in TREATgermany, 134 patients received CSA. Conclusion: With the increasing number of recruitment centers (October 2020: 38 centers; May 2021: 59 centers), TREATgermany can continue to make an important contribution to health services research for patients with moderate-to-severe AD. The registry fulfills the methodological requirements of IQWiG for the collection and processing of healthcare-related data. With the successful and expected approval of further systemic treatments, these can be compared in terms of efficacy and safety in the future. In addition, with the recruitment of children and adolescents started in 2021, this patient group can also be observed.

## Introduction 

Atopic dermatitis (AD, synonym: atopic eczema) is a chronic inflammatory, often episodic dermatosis affecting ~ 8% of adults and ~ 7% of children and adolescents in Germany [1, 2, 3]. A previous multinational epidemiological study in adults reported a cross-national prevalence of 2.1 – 4.9% [1]. In order to monitor moderately to severely affected children, adolescents, and adults with AD in Germany with regard to the clinical and subjective course of the disease as well as the course of therapy, the German registry TREATgermany collects comprehensive parameters for disease monitoring. 

In 2016, TREATgermany emerged from TREATeczema and today represents a high-quality national registry that fulfills all quality criteria and methodological requirements for clinical registries as described in the Memorandum Register for Health Services Research [4] and in the criteria of the Institute for Quality and Efficiency in Health Care (IQWiG, Rapid Report) [5]. 

Starting in 2016, adult patients have been observed in TREATgermany and, since January 2021, children and adolescents have also been recruited. The study design of a non-interventional, prospective cohort study collects data from routine health care data. The detailed description of the registry, including the variables collected, the baseline characteristics of the first 612 adult patients, and analyses of reduced quality of life have been reported previously [6, 7]. 

In the following, we report the current status and results of the October 2020 interim data analysis of the TREATgermany registry. 

## Materials and methods 

TREATgermany recruits patients with moderate-to-severe AD according to the diagnostic criteria of the UK Working Party [8], i.e., an oSCORAD (“Objective Scoring for Atopic Dermatitis”) [9] > 20 [10] and/or an systemic therapy for AD within the past 24 months before inclusion in the registry. No study related interventions will be performed. Included patients will be followed up prospectively for at least 24 months. During the follow-up period, standardized study visits will be conducted to prospectively document patient characteristics, clinical data and symptomatology, patient-reported or subjective disease severity, clinical reasons for treatment decisions, and treatment satisfaction. 

Currently, 59 dermatological practices, clinics, and university hospitals are participating in TREATgermany (Table 1). 

The main objectives of TREATgermany are:

- To describe the medical care and pharmaceutical therapies of patients suffering from moderate-to-severe AD; to focus on patients’ perspectives on benefits, treatment goals, quality of life, and treatment satisfaction, and on the sequence of treatments;
- To provide health care data for the comparative efficacy, tolerability, and safety of systemic therapies for moderate-to-severe AD;
- To provide a platform, network, and other resources for basic molecular research and embedded randomized clinical trials.

In accordance with the recommended Core Outcome Set (COS) of the Harmonizing Outcome Measures for Eczema Initiative (HOME Initiative) [11, 12, 13], the target parameters for evaluating the effectiveness of treatments for AD include:

- clinical symptom severity (“Eczema Area and Severity Index” score (EASI) [14], oSCORAD)
- subjective symptom severity (“Patient-Oriented Eczema Measure” (POEM) [15, 16], itching and sleep disturbances)
- disease episodes (total number/well-controlled weeks)
- and quality of life (“Dermatological Quality of Life Index” (DLQI) [17, 18]).

Furthermore, side effects, adverse events and reasons for treatment discontinuation are documented by the recruiting centers. Patient and physician satisfaction with treatment and reasons for choosing certain interventions are assessed. 

Data collection is conducted in an online database and electronic patient questionnaires. 

The TREATgermany research project was submitted to all relevant ethics committees and received a positive vote (No. EK TUD 118032016). TREATgermany is registered in the clinicatrials.gov database (NCT03057860) and the ENCePP Resource Database (EMA). 

## Results 

**Figure 1.**
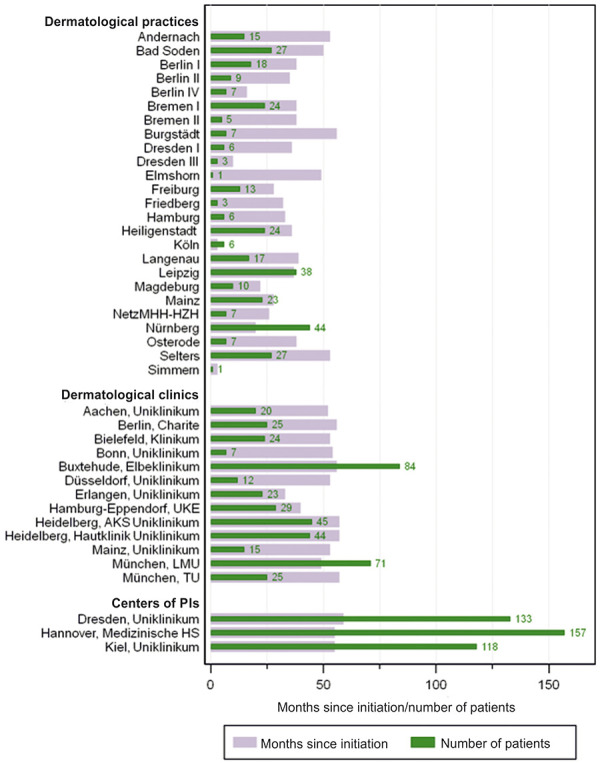
Recruited adult patients in TREATgermany (as of May 25, 2021).

**Figure 2.**
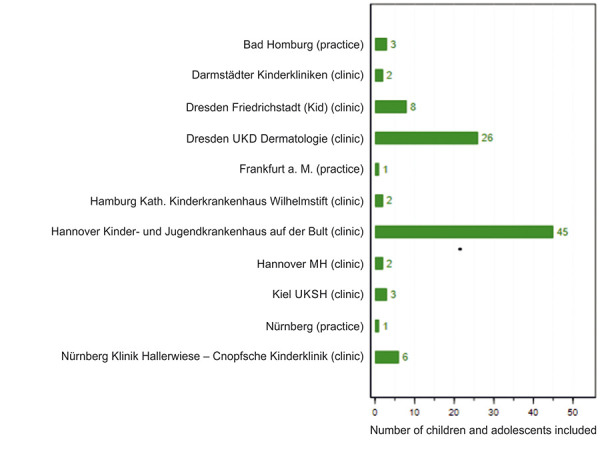
Recruited children and adolescents in TREATgermany (as of May 25, 2021).

TREATgermany has included 1,180 adults and 99 children or adolescents in the registry by May 2021 (Figures 1, 2). 

The following data descriptions are based on the October 13, 2020, interim analysis and refer to 1,025 enrolled adult patients with 4,373 documented visits from 38 recruitment centers at that time. 

### Characteristics of the included patients 

The mean age at inclusion in the registry was 42 years, 57.7% of patients were men (n = 591), and 42.3% were women (n = 434). According to oSCORAD, 85.8% of the included patients were moderately to severely affected by AD. More than half of the patients were affected on the face (78.0%), hands (74.6%), large joint flexures (76.1%), or neck (78.0%). 

The majority of patients stated that they lived in a partnership. 77.6% of patients were employed, and of these 73.2% in full-time. 50.9% reported that they had not previously smoked. At the baseline visit, patients were also asked about their medical history regarding AD. 57.0% of registry patients stated that they had AD since infancy. 13.9% and 10.7%, reported onset of AD before/at school age. 52.5% of patients being employed reported sickness absence caused by AD in the year before enrollment for registry (median: 14 days/year). 43.6% and 23.8% of patients reported hospitalization and rehabilitation due to AD in the 5 years prior to inclusion. 

65.9% of patients also reported that their skin was permanently affected by AD in the year prior to inclusion in the registry. The remaining 34.1% of patients were affected by AD for an average of 6 months (6.3 ± 3.2) of the past year. The percentage of body surface area (BSA) affected within the last 3 days before the visit was 19.0% on average (19.0 ± 22.5, median = 11), according to the patients’ assessment. 

Regarding their concomitant diseases, 17.9% and 17.8% of the patients stated that they had suffered from allergic rhinitis and allergic asthma, in the past. Another 52.1% and 33.4% of the patients reported corresponding symptoms in the past year. In summary, 70.0% and 51.2% of patients reported suffering from allergic rhinitis and allergic asthma. 

At the baseline visit, ~ 40% of patients reported that their AD was severe or very severe. More than half experienced an extremely or very large effect on their quality of life (according to DLQI). The POEM sum score as a subjective reflection of disease severity is consistent with this finding. In contrast, ~ 60% of patients with AD did not expirienced increased fatigue. The same percentage reported no or only mild symptoms of depression. Nevertheless, ~ 40% of those affected by AD showed fatigue and depressive symptoms. 

The detailed baseline characteristics of the 1,025 patients and their clinical and subjective symptom severity are shown in Tables 2, 3, and 4. 

The most frequently reported subjective symptoms of AD were pruritis, pain, and sleep disturbance. Using 11-point Likert scales (0 = no symptom to 10 = most severe symptoms imaginable), patients rated pruritus as the most severe (mean 5.7), followed by sleep disturbance (mean 4.5) and pain (mean 3.7). 

Patients perceived their AD as well controlled for an average of 4.4 (4.4 ± 3.8) weeks of the past 12 weeks. The mean number of weeks perceived as fully controlled was 2 weeks (2.0 ± 3.4). The percentage of patients reporting no weeks without (fully controlled) symptoms within the last 12 weeks was 57.7%. 

### Systemic therapy of TREATgermany patients 

At the time of inclusion in TREATgermany, 600 patients had already received systemic therapy with glucocorticosteroids, 307 with ciclosporin A, 45 with methotrexate, 28 with azathioprine, and 98 with dupilumab in the course of their disease history. Lower numbers of cases were documented for mycophenolates, alitretinoin, JAK inhibitors, and other systemic treatments (Table 5). 91.3% (n = 927) of patients received topical glucocorticosteroids, and 38.0% (n = 381) received UV therapy in the 12 months before inclusion in the registry. Based on the data from October 2020 including all follow-up visits, 597 patients received systemic therapy with dupilumab during their participation in TREATgermany, 442 had this therapy initated during their participation in the registry (40 of them outside registry visits). 134 patients received systemic therapy with ciclosporin A during their participation in TREATgermany. 

The subgroup of patients who received dupilumab therapy showed a better improvement of the symptoms compared to patients who did not receive systemic therapy. The mean oSCORAD for dupilumab patients at the 3^rd^ follow-up visit was 19.9 (SD 9.9) and for patients not receiving systemic therapy was 27.7 (SD 13.6). The EASI score showed the same trend (dupilumab mean 4.1 (SD 4.4) vs. no systemic therapy mean 7.0 (SD 7.0)). The results of subjective symptom severity, measured by POEM score, pain, pruritus and sleep disturbance, disease relapses, and Patient Global Assessment (PGA), also showed a greater improvement for the dupilumab patients. These comparative follow-up studies were performed only for individuals who were initiated on dupilumab during their participation in TREATgermany and who had at least three documented follow-up visits (n = 224, including at least one follow-up visit with dupilumab therapy). Patients who were not prescribed systemic therapy either at enrollment or at the first three follow-up visits were selected as the comparison group (n = 129). 

### Adverse events 

Adverse events occurred in 246 registry patients (24%) in 407 visits, with 462 individual adverse events documented. The most frequently mentioned adverse event was conjunctivitis (n = 227; of which n = 222 in patients with dupilumab therapy), as well as skin disorders (n = 24), headache (n = 20), herpes disorders (n = 19), eye disorders (not conjunctivitis, n = 19), and gastrointestinal complaints (n = 18). All other adverse events occurred less frequently (n ≤ 15) (Table 6). 

## Discussion 

As a prospective cohort study, TREATgermany makes an important contribution to health services research on the treatment of AD in Germany. In addition, TREATgermany is part of the European registry family TREAT [19], which should enable the pooling of data for safety and efficacy analyses from different European countries in the future. 

The reported interim analysis of the patients of the TREATgermany registry of October 13, 2020 characterized the 1,025 adults enrolled in the registry up to that date with regard to demographic values, previous diseases, clinical disease severity, the subjective disease severity of the patients, the applied systemic and concomitant therapies (e.g., topical therapies). 

The population under consideration consisted of slightly more men (57.7%) than women (42.3%) and was on average 42 years old. More than half of the patients lived in a stable partnership (married and unmarried, 64.8%), ~ 50% had a high level of education (high school diploma, vocational diploma, or university degree), and 73.2% were employed full-time. The observed registry patients were affected by the comorbidities associated with AD [2] – allergic rhinoconjuctivitis and bronchial asthma (65.9% and 44.3%). Other common comorbidities were arterial hypertension (18.8%) and depression (9.7%). The association of AD and depression is in line with previous research findings and higher than in the general population of Germany (9.7 vs. 7.7%) [20, 21]. The association between AD and other autoimmune diseases reported in the literature [22] could not be observed in the previous data of the TREATgermany registry population. 

In terms of clinical symptom severity, the adult patients were predominantly moderately to severely affected by AD (oSCORAD 85.8%, EASI score 76.8%). Face, hands, and the large joints were affected in more than 70%. The patients themselves rated their symptom severity similarly severe (POEM sum score 86.0%). Consequently, more than half also experienced a severe or very severe limitation with regard to their quality of life (DLQI 51.0%). The results regarding subjective symptom severity and impaired quality of life are consistent with previous scientific findings [23]. The percentage of patients with moderate AD (Investigator Global Assessment (IGA) 21.9% for the categories clear/almost clear/mild) can be explained by the fact that patients with ongoing or previous systemic therapy could also be included in the registry. 

According to the inclusion criteria for TREATgermany, a high proportion of patients (n = 744) already received systemic therapy. Based on the data from October 2020, 597 patients received systemic therapy with dupilumab during their participation in the registry, 442 were prescribed this therapy during their participation in the registry. This high proportion is due to the limited treatment options available at that time. The subgroup of patients receiving dupilumab therapy showed a better clinical and subjective response compared to patients who did not receive systemic therapy. 

## Conclusion and outlook 

With the increasing number of recruitment centers (October 2020: 38 centers, May 2021: 59 centers), TREATgermany can continue to make an important contribution to health services research for patients with moderate-to-severe AD and fulfills the methodological requirements of IQWiG for the collection and processing of health services-related data [5]. In addition to the description of various parameters from the perspective of clinicians and patients, a comprehensive picture of the current health care situation in Germany can be obtained through the recruitment from dermatological practices, clinics, and university hospitals. With the successful and expected approval of further systemic therapies since October 2020 (for example baricitinib [24, 25, 26], tralokinumab [27, 28], upadacitinib [29, 30]), further reports from the TREATgermany registry can also analyze interesting comparisons regarding clinical and subjective parameters, investigate the occurrence of side effects and compare them for different therapy options. 

In January 2021, the registry was expanded to include children and adolescents. Until June 2021, 18 centers have been initiated and 99 children and adolescents have been included. A special feature of the survey of subjective symptom severity by the patients is the linguistic adaptation of various standardized questionnaires according to the age of the children and adolescents. First results for children and adolescents suffering from moderate-to-severe AD will be reported in 2022. 

The collection of routine health care data through the TREATgermany registry and the expansion to all age groups will provide a better understanding of moderate-to-severe AD to all those involved in treatment and to those affected. All dermatology clinics, outpatient dermatologists, and general practitioners with a focus on AD in Germany are invited to participate as recruitment centers. 

## Acknowledgment 

The TREATgermany study group consists of the centers named in the list of authors and the following recruiting centers:

- S. Abraham: Clinic and Polyclinic for Dermatology, Carl Gustav Carus University Hospital and Medical Faculty at the Technical University of Dresden.
- M. Asefi: Dermatological Study Center Hunsrück, Simmern.
- A. Asmussen: Practice Andrea Asmussen, MD, dermatology at the Lesum, Bremen.
- M. Augustin: Institute for Health Services Research in Dermatology and Nursing Professions, University Medical Center Hamburg-Eppendorf.
- M. Bell: Practice Magnus Bell, MD, Andernach.
- T. Bieber: Clinic and Polyclinic for Dermatology and Allergology, University Hospital Bonn.
- T. Biedermann: Clinic for Dermatology and Allergology, Klinikum rechts der Isar, Technical University of Munich.
- U. Boashi: Dermatology practice Ute Boashie, MD, Dresden.
- JJ. Brücher: Dermatology Outpatient Clinic Magdeburg.
- P. Buck: Practice Phillipp Buck, MD, Goldbek Medical Dermatology, Hamburg.
- R. Döllmann: Pediatric practice Dr. med. Robert Döllmann, Dresden.
- I. Effendy: Department of Dermatology, Rosenhöhe Clinic, Bielefeld.
- K. Ertner: Practice Dr. med. Konstantin Ertner, Nuremberg.
- K. Gardlo: Joint practice Dr. med. Kerstin Gardlo/Dr. med. Anette Jovic-Paris, Bad Neuenahr.
- B. Gerlach: Practice Dr. med. Beatrice Gerlach, Dresden.
- H. Gorriahn-Maiterth: Dermasana Practice, Karlsruhe.
- B. Großmann: Dermatology practice of Dr. Bernd Großmann, Koblenz.
- E. Hamelmann: Clinic for Pediatric and Adolescent Medicine, Evangelisches Klinikum Bethel, Bielefeld.
- C. Handrick: Practice Christiane Handrick, MD, Berlin.
- U. Heimann: Dermatology practice Ulrike Heimann, Papenburg-Aschendorf.
- P. Höger: Pediatrics and Pediatric Dermatology/Allergology, Kath. Kinderkrankenhaus Wilhelmstift, Hamburg.
- M. Hoffmann: Practice Dr. med. Matthias Hoffmann, Witten.
- B. Homey: Clinic for Dermatology, University Hospital Düsseldorf.
- S. Kerzel: Barmherzige Brüder Hospital Regensburg, WECARE Study Center, KUNO Clinic St. Hedwig, Regensburg.
- R. von Kiedrowski: CMSS – Company for Medical Study and Service, Selters/Westerwald.
- A. Kleinheinz: Clinic for Dermatology, Allergology Outpatient Clinic, Elbe Klinikum Buxtehude.
- S. Lau: Section Pediatric Pneumology/Allergology/Endoscopy, Charité Universitätsmedizin Berlin.
- M. Mempel: Dermatology practice Prof. Dr. med. Martin Mempel, Elmshorn.
- K. Nemat: Practice for Pediatric Pneumology and Allergology, Children’s Center Dresden-Friedrichstadt.
- K. Neubert: Practice Dipl.-Med. Kathrin Neubert, Burgstädt.
- I. Neustädter: Cnopfsche Kinderklinik/Neonatologie, Pädiatrie, DIAKONEO KdöR, Nuremberg.
- H. Ott: Pediatric Dermatology and Allergology, Kinder- und Jugendkrankenhaus auf der Bult, Hannover.
- M. Pawlak: Practice Dr. med. Anika Hünermund and Mario Pawlak, Heilbad Heiligenstadt.
- A. Pinter: Department of Dermatology, Venereology and Allergology, Clinical Research, University Hospital Frankfurt am Main.
- I. Reitenbach-Blindt: Dermasana practice, Eggenstein-Leopoldshafen.
- J. Rossbacher: Practice Jens Rossbacher/ Dr. med. Klaus Spickermann, Hautzentrum Friedrichshain, Berlin.
- R. Salgo: Pediatric Dermatology Practice Dr. med. Rebekka Salgo, Frankfurt am Main.
- T. Schaefer: Practice Dr. med. Thomas Schaefer/Dr. med. Doreen Belz, Derma Cologne.
- K. Schäkel: University Department of Dermatology, Heidelberg University Hospital.
- F. Schenck: Dermatology Center, Hannover.
- B. Schwarz: Practice Dr. med. Beate Schwarz, Dermatology and Allergology, Langenau.
- U. Schwichtenberg: Derma-nord Dermatology practices, Bremen.
- T. Schirmer: Practice Dr. med. Thomas Schirmer, Berlin.
- M. Stahl: Practice Maren Stahl, M.D., Osterode.
- P. Staubach-Renz: University Department of Dermatology and Polyclinic, University Medical Center Mainz.
- S. Quist: Joint practice of Prof. Dr. Dr. med.
- M. Sticherling: Department of Dermatology, Erlangen University Hospital.
- H. Straube: Darmstädter Kinderkliniken Prinzessin Margaret, Darmstadt.
- C. Vogelberg: Department of Pediatrics and Adolescent Medicine, University Hospital Carl Gustav Carus Dresden.
- S.H. Hong-Weldemann: Practice Sung-Hei Hong-Weldemann, M.D., Freiburg.
- C. Walter: Joint practice Dr. med. Christian Walter, Bad Homburg.
- F. Wiemers: Practice Dr. med. Franca Wiemers, Leipzig.
- J. Wildberger: Practice Dr. med. Julia Wildberger, Dermatology, Bad Soden.
- T. Wildfeuer: Practice Dr. med. Thomas Wildfeuer, Berlin.
- A. Wollenberg: Clinic and Polyclinic for Dermatology and Allergology, LMU Munich.
- E. Weisshaar: Occupational Dermatology, Department of Dermatology, Heidelberg University Hospital.
- M. Worm: Clinic for Dermatology, Venerology and Allergology at Campus Mitte (CCM), Charité Universitätsmedizin Berlin.

## Funding 

TREAT Germany is an academic, investigator-initiated clinical registry financially supported by Sanofi-Aventis Deutschland GmbH, Galderma S.A., LEO Pharma GmbH, Lilly Deutschland GmbH, and Abbvie. 

## Conflicts of interest 

TW is Co-Principal Investigator of the German Atopic Dermatitis Registry TREATgermany. TW has received honoraria for lectures or scientific advice on atopic dermatitis from AbbVie, Almirall, Galderma, Jans-sen/JNJ, Leo Pharma, Leti, Lilly, Novartis, Pfizer, and Regeneron/Sanofi. 

SW is Co-Principal Investigator of the German Atopic Dermatitis Registry TREATgermany. SW has received institutional research grants from Novartis, L’Oreal, and La Roche Posay; he has performed consulting work for Sanofi-Genzyme, Regeneron, LEO Pharma, Eli Lilly, AbbVie, Pfizer, GSK, and Kymab; he has also lectured at educational events sponsored by Sanofi-Genzyme, Regeneron, LEO Pharma, Eli Lilly, Ab-bVie, and Pfizer; and received travel grants from Sanofi-Genzyme, Regeneron, LEO Pharma, Eli Lilly, AbbVie, and Pfizer. 

JS is leading Principal Investigator of the German Atopic Dermatitis Registry TREATgermany. JS has received institutional grants from Novartis and Pfizer for scientifically initiated research and honoraria for consulting from Sanofi, Lilly, Novartis, and ALK. 

All other authors declare no conflict of interest. 

**Table 1.** Centers of the TREATgermany registry (as of May 2021).

**Centers that recruit adults only (41)**
- Praxis Dr. med. Magnus Bell/Dr. med. Thomas Kaiser, Andernach Gemeinschaftspraxis - Dr. med. Kerstin Gardlo/Dr. med. Anette Jovic-Paris, Bad Neuenahr Praxis - Dr. med. Julia Wildberger, Hautmedizin, Bad Soden - Prof. Dr. med. Margitta Worm, Klinik für Dermatologie, Venerologie und Allergologie, Allergiezentrum, Charité Universitätsmedizin Berlin Praxis - Dr. med. Christiane Handrick, Berlin - Praxis Jens Rossbacher/Dr. med. Klaus Spickermann, Hautzentrum Friedrichshain, Berlin Praxis - Dr. med. Thomas Wildfeuer, Berlin - Praxis Dr. med. Thomas Schirmer, Berlin - Prof. Dr. med. Isaak Effendy, Hautklinik des Klinikums Rosenhöhe, Bielefeld - Prof. Dr. med. Dr. ès sci. Thomas Bieber, Klinik und Poliklinik für Dermatologie und Allergologie, Universitätsklinikum Bonn - Praxis Dr. med. Uwe Schwichtenberg, Hautpraxen Derma-nord, Bremen - Praxis Dr. med. Andrea Asmussen, Dermatologie an der Lesum, Bremen - Praxis Dipl.-Med. Kathrin Neubert, Burgstädt - Praxis Dr. med. Beatrice Gerlach, Dresden - Hautarztpraxis Dr. med. Ute Boashie, Dresden - Prof. Dr. med. Bernhard Homey, Klinik für Dermatologie, Universitätsklinikum Düsseldorf - Ina Reitenbach-Blindt, Praxis Dermasana, Eggenstein-Leopoldshafen Hautarztpraxis - Prof. Dr. med. Martin Mempel, Elmshorn - Dr. med. Andreas Pinter, Klinik für Dermatologie, Venerologie und Allergologie, Klinische Forschung, Universitätsklinikum Frankfurt am Main - Hautarztpraxis Dr. med. Sung-Hei Hong-Weldemann, Freiburg - Prof. Dr. med. Matthias Augustin, Institut für Versorgungsforschung in der Dermatologie und bei Pflegeberufen, Universitätsklinikum Hamburg-Eppendorf - Praxis Dr. med. Phillipp Buck, Goldbek Medical Dermatologie, Hamburg - Dr. med. Florian Schenck, Hautärzte Zentrum, Hannover - Prof. Dr. med Elke Weisshaar, Berufsdermatologie, Hautklinik, Universitätsklinikum Heidelberg - Prof. Dr. med. Knut Schäkel, Universitäts-Hautklinik, Universitätsklinikum Heidelberg - Praxis Dr. med. Anika Hünermund/Mario Pawlak, Heilbad Heiligenstadt - Praxis Hannah Gorriahn-Maiterth, Praxis Dermasana, Karlsruhe - Praxis Dr. med. Thomas Schaefer/Dr. med. Doreen Belz, Derma Köln - Hautarztpraxis Dr. med. Bernd Großmann, Koblenz - Praxis Dr. med. Beate Schwarz, Dermatologie und Allergologie, Langenau - Praxis Dr. med. Franca Wiemers, Leipzig - Praxis Dr. med. Jens-Joachim Brücher, Hautambulatorium Magdeburg - Prof. Dr. med. Petra Staubach-Renz, Universitäts-Hautklinik und Poliklinik, Universitätsmedizin Mainz - Gemeinschaftspraxis Prof. Dr. Dr. med. Sven Quist, Mainz - Prof. Dr. med. Tilo Biedermann, Klinik für Dermatologie und Allergologie, Klinikum rechts der Isar, TU München - Prof. Dr. med. Dr. h.c. Andreas Wollenberg, Klinik und Poliklinik für Dermatologie und Allergologie, LMU München - Praxis Dr. med. Maren Stahl, Osterode - Hautarztpraxis Ulrike Heimann, Papenburg-Aschendorf - Praxis Dr. med. Ralph von Kiedrowski, Company for Medical Study and Service Selters, Selters - Dr. med. Mohammad Asefi, Dermatologisches Studienzentrum Hunsrück, Simmern - Praxis Dr. med. Matthias Hoffmann, Witten
**Centers that recruit only children (12)**
- Gemeinschaftspraxis Dr. med. Christian Walter, Bad Homburg Prof. Dr. med. Susanne Lau, Sektion Pädiatrische Pneumologie/Allergologie/Endoskopie, Charité Universitätsmedizin Berlin - Prof. Dr. Eckard Hamelmann, Klinik für Kinder- und Jugendmedizin, Evangelisches Klinikum Bethel, Bielefeld - Dr. med. Helen Straube, Darmstädter Kinderkliniken Prinzessin Margaret, Darmstadt - Prof. Dr. med. Christian Vogelberg, Klinik für Kinder- und Jugendmedizin, Universitätsklinikum Carl Gustav Carus Dresden - Dr. med. Katja Nemat, Praxis für Kinderpneumologie und Allergologie, Kinderzentrum Dresden-Friedrichstadt - Kinderarztpraxis Dr. med. Robert Döllmann, Dresden - Kinderhautarztpraxis Dr. med. Rebekka Salgo, Frankfurt am Main - Prof. Dr. med. Peter Höger, Pädiatrie und Pädiatrische Dermatologie/Allergologie, Kath. Kinderkrankenhaus Wilhelmstift, Hamburg - PD Dr. med. Hagen Ott, Pädiatrische Dermatologie und Allergologie, Kinder- und Jugendkrankenhaus auf der Bult, Hannover - Dr. med. Irena Neustädter, Cnopfsche Kinderklinik / Neonatologie, Pädiatrie, DIAKONEO KdöR, Nürnberg - PD Dr. med. Sebastian Kerzel, Krankenhaus Barmherzige Brüder Regensburg, WECARE Studienzentrum, KUNO Klinik St. Hedwig, Regensburg
**Centers that recruit adults and children (6)**
- Dr. med. Andreas Kleinheinz, Klinik für Dermatologie, Allergologische Ambulanz, Elbe Klinikum Buxtehude - Dr. med. Susanne Abraham, Klinik und Poliklinik für Dermatologie, Universitätsklinikum und Medizinische Fakultät Carl Gustav Carus an der Technischen Universität Dresden - Prof. Dr. med. Michael Sticherling, Hautklinik, Universitätsklinikum Erlangen - Prof. Dr. med. Thomas Werfel, Abteilung Immundermatologie und experimentelle Allergologie, Klinik für Dermatologie, Allergologie und Venerologie, Medizinische Hochschule Hannover - Prof. Dr. med. Stephan Weidinger, Klinik für Dermatologie, Venerologie und Allergologie, Universitätsklinikum Schleswig-Holstein, Campus Kiel Praxis - Dr. med. Konstantin Ertner, Nürnberg
**Centers that left TREATgermany (as of December 2020) (2)**
- Dr. med. Melanie Hilgers, Klinik für Dermatologie und Allergologie, Hautklinik, Universitätsklinikum Aachen - Dr. med. Ekaterin a Tchitcherina, Praxis für Haut-und-Geschlechtskrankheiten, Friedberg

**Table 2.** Overview of demographic characteristics of all patients at the baseline visit (n = 1,025, discrepancies in totals for the different variables are due to missing data).

Baseline characteristics	Number of patients
Sex	
Male	591 (57.7%)
Female	434 (42.3%)
Age, mean (SD)	41.7 (14.6) (n = 1,025)
BMI, mean (SD)	25.9 (5.4) (n = 1,009)
Marital status	
Solid partnership, unmarried	265 (26.2%)
Married	391 (38.6%)
Divorced	42 (4.2%)
Widowed	11 (1.1%)
Single	303 (29.9%)
Education level	
Without school-leaving qualification	8 (0.8%)
Lower secondary school graduates	124 (12.2%)
Secondary school diploma	377 (37.2%)
High school diploma	259 (25.5%)
University degree	246 (24.3%)
Gainful employment	
Not employed	227 (22.4%)
Employed	788 (77.6%)
Employment status	
Full-time (35 hours and more)	574 (73.2%)
Part time or hourly	172 (21.9%)
Leave of absence (parental leave or similar)	8 (1.0%)
Trainee, re-trainee	30 (3.8%)
Non-employed persons	
Pensioner/retiree in early retirement	71 (32.0%)
Househusband/Housewife	24 (10.8%)
Pupil/student	67 (30.2%)
Unemployed	46 (20.7%)
Early retiree	14 (6.3%)
Smoker status	
Smoker	246 (24.2%)
Ex-smoker (< 10 years not smoked)	143 (14.1%)
Ex-smoker (≥ 10 years not smoked)	109 (10.7%)
Never smoked	517 (50.9%)
Allergic comorbidities	
Allergic rhinoconjunctivitis (hay fever)	650 (65.9%)
Bronchial asthma	443 (44.3%)
Other comorbidities	
Arterial hypertension	189 (18.8%)
Heart failure (≥ NYHA III)	5 (0.5%)
Previous myocardial infarction (queried since 2018)	7 (0.9%)
Previous Apoplex (queried since 2018)	4 (0.5%)
Diabetes mellitus type 1	1 (0.1%)
Diabetes mellitus type 2	36 (3.5%)
Crohn’s disease/Colitis ulcerosa	12 (1.2%)
Renal failure	11 (1.1%)
Rheumatoid arthritis	3 (0.3%)
Depression	97 (9.7%)
Cancers (queried since 2018)	16 (2.0%)

SD = standard deviation.

**Table 3.** Clinical symptom severity and body regions affected at baseline visit (n = 1,025, discrepancies in totals for different variables are due to missing data).

Clinical symptom severity	Number of patients
IGA	
Clear	15 (1.5%)
Almost clear	61 (6.0%)
Mild	146 (14.4%)
Moderate	400 (39.4%)
Severe	314 (30.9%)
Very severe	79 (7.8%)
oSCORAD	
oSCORAD, mean (SD)	40.9 (16.2) (n = 1,016)
oSCORAD – Categories	
Clear (0 – < 8)	26 (2.6%)
Mild (8 – < 24)	118 (11.6%)
Moderate (24 – < 38)	295 (29.0%)
Severe (38 – 83)	577 (56.8%)
EASI	
EASI, mean (SD)	16.1 (12.9) (n = 1,015)
EASI – Categories	
Clear (0)	14 (1.4%)
Mild (>0 – <6)	221 (21.8%)
Moderate (6 – <23)	539 (53.1%)
Severe (23 – 72)	241 (23.7%)
Affected body regions	Number of patients
	n = 1,015
Face	792 (78.0%)
Hands	757 (74.6%)
Feet	435 (42.9%)
Genital area	155 (15.3%)
Large joint flexions (queried since 2018, *n*_total_ * = 762*)	580 (76.1%)
Neck (queried since 2018, *n*_total_ * = 762*)	594 (78.0%)

SD = standard deviation.

**Table 4.** Subjective symptom severity at baseline visit (n = 1,025, discrepancies in totals for different variables are due to missing data).

Subjective symptom severity	Number of patients
PGA	
Clear	26 (2.6%)
Almost clear	81 (8.0%)
Mild	207 (20.5%)
Moderat	299 (29.5%)
Severe	287 (28.4%)
Very severe	112 (11.1%)
DLQI	
DLQI, mean (SD)	11.8 (7.8) (n = 1,012)
DLQI – Categories	
No effect at all on patient’s life (0 – 1)	85 (8.4%)
Small effect on patient’s life (2 – 5)	179 (17.7%)
Moderate effect on patient’s life (6 – 10)	232 (22.9%)
Very large effect on patient’s life (11 – 20)	356 (35.2%)
Extremely large effect on patient’s life (21 – 30)	160 (15.8%)
POEM	
POEM, mean (SD)	16.8 (7.6) (n = 1,014)
POEM – Categories	
Clear or almost clear (0 – 2)	50 (4.9%)
Mild (3 – 7)	93 (9.2%)
Moderate (8 – 16)	316 (31.2%)
Severe (17 – 24)	375 (37.0%)
Very severe (25 – 28)	180 (17.8%)
Fatique, mean (SD)	3.7 (1.6) (n = 1,011)
Fatique	
Fatique – Categories	
Normal (FSS ≤ 4)	613 (60.6%)
Increased (FSS > 4 and ≤ 5)	163 (16.1%)
High (FSS > 5)	235 (23.2%)
CESD-D	
CESD-D, mean (SD)	14.9 (10.1) (n = 1,014)
CESD-D – Categories	
No to mild depressive symptoms (0 to < 16)	616 (60.7%)
Moderate depressive symptoms (≥ 16 to < 24)	208 (20.5%)
Major depressive symptoms (≥ 24)	190 (18.7%)

SD = standard deviation.

**Table 5.** Systemic therapy for atopic dermatitis before inclusion in the registry (n = 1,021; all patients for whom physicians provided information on systemic treatment before inclusion. For each substance, the response category “no” and lack of response was considered as no specific therapy before inclusion. The response category “unclear” was considered as missing information, and these patients were excluded from the respective population. Some of the other systemic therapies were administered within (blinded) trials).

Substance	n_total_	Number of patients with previous therapy
Glucocorticosteroids	1,009	600 (59.5%)
Ciclosporin A	1,013	307 (30.3%)
Methotrexate	1,014	45 (4.4%)
Azathioprine	1,015	28 (2.8%)
Mycophenolate	1,015	21 (2.1%)
Dupilumab (queried since 2018)	1,015	98 (9.7%)
Other systemic therapies	1,015	91 (9.0%)
Alitretinoin		24 (2.4%)
JAK inhibitor (baricitinib)		4 (0.4%)
JAK inhibitor (upadacitinib)		1 (0.1%)
JAK inhibitor (unspecified)		2 (0.2%)
Secukinumab		3 (0.3%)
Tralokinumab anti-IL13-AK		8 (0.8%)
Other (including unspecified study medications)		49 (4.8%)

**Table 6.** Documented adverse events in TREATgermany (as of October 13, 2020).

Adverse event	Number of patients
Conjunctivitis	227
Skin diseases	24
Headache	20
Herpes diseases	19
Eye diseases (other than conjunctivitis)	19
Gastrointestinal complaints	18
Colds	14
Flu-like symptoms	11
Hair loss	11
Orthopedic conditions	11
Fatigue	10
Joint diseases	10
Discomfort at the injection site	7
Weight gain	7
Cardiovascular diseases	7
Dizziness	4
Migraine	3
Menopausal symptoms	3
Cystitis	2
Depression	2
Hair growth	2
Kidney diseases	2
Sleep disorders	2
Tumor diseases (other than dermatological)	2
Gingival disease	2
Apoplexy	2
Respiratory diseases	1
Lyme disease	1
Corona infection SARS-CoV-2 infection	1
Eczema	1
Heart attack	1
Hearing loss	1
Circulatory disorders	1
Liver disease	1
Pain	1
Sweating	1
Blood formation disorders	1
Edema	1
Other (not specified)	9
